# Morphological and histological study of the forewing of *Orthezia urticae* (Linnaeus, 1758) (Hemiptera, Sternorrhyncha)

**DOI:** 10.3897/zookeys.747.23950

**Published:** 2018-04-02

**Authors:** Barbara Franielczyk-Pietyra, Łukasz Depa, Piotr Wegierek

**Affiliations:** 1 Department of Zoology, Faculty of Biology and Environmental Protection, University of Silesia, Bankowa 9, 40-007 Katowice, Poland

**Keywords:** Coccoids, cross-section, forewing, morphology, ultrastructure, wing veins

## Abstract

Wings of *Orthezia urticae* males were studied. Both ventral and dorsal surfaces of wings were examined under light and scanning electron microscopes. The structure regarded as vein cubitus anterior turned out to be a reinforcement element only. Two elements known as radius sector and media are almost transparent depressions in the wing membrane. Veins at the margin of the fold of the wing anal lobe were not confirmed. Studies indicated a row of sensilla cupola at the beginning of the subcostal ridge.

Cross sections of the wing membrane showed a two-layered membrane. The presence of two veins was confirmed in a common stem – subcostal and radius. The change of common stem shape was described. Neither tracheae nor nerves were observed. This is the second paper on cross-sections of wing within Sternorrhyncha.

## Introduction

Infraorder Coccomorpha (Fallen 1814) comprises scale insects, also known as coccoids. They are sap-sucking hemipterans, divided into two main groups: more primitive archaeococcoids and “advanced” neococcoids (Gullan and Martin 2009). The genus *Orthezia* belongs to the first group (family Ortheziidae) and has the wings regarded as the most primitive within known scale insects because they retain all fundamental elements of venation (Shcherbakov 2007). However, on the basis of current studies of recent and fossil species, the family Ortheziidae does not seem to be as primitive as previously thought (Vea and Grimaldi 2012).

The analysis of the *O. urticae* forewing was made in detail by Koteja (1986) and partly by Shcherbakov (2007). The distinctive feature of coccoid females is the wing loss, which is complete and no organs replace them. It is most probably related to the parasitic life mode, i.e. life on host plant and feeding on it (Koteja 1985). In males, forewings are folded flat over the abdomen and overlap each other (Brodsky 1996), while hindwings are reduced to hamulohalteres (Koteja 1986). However, venation is strongly reduced in this insect group and the nomenclature of the veins is still highly questionable.

**Figure 1.**
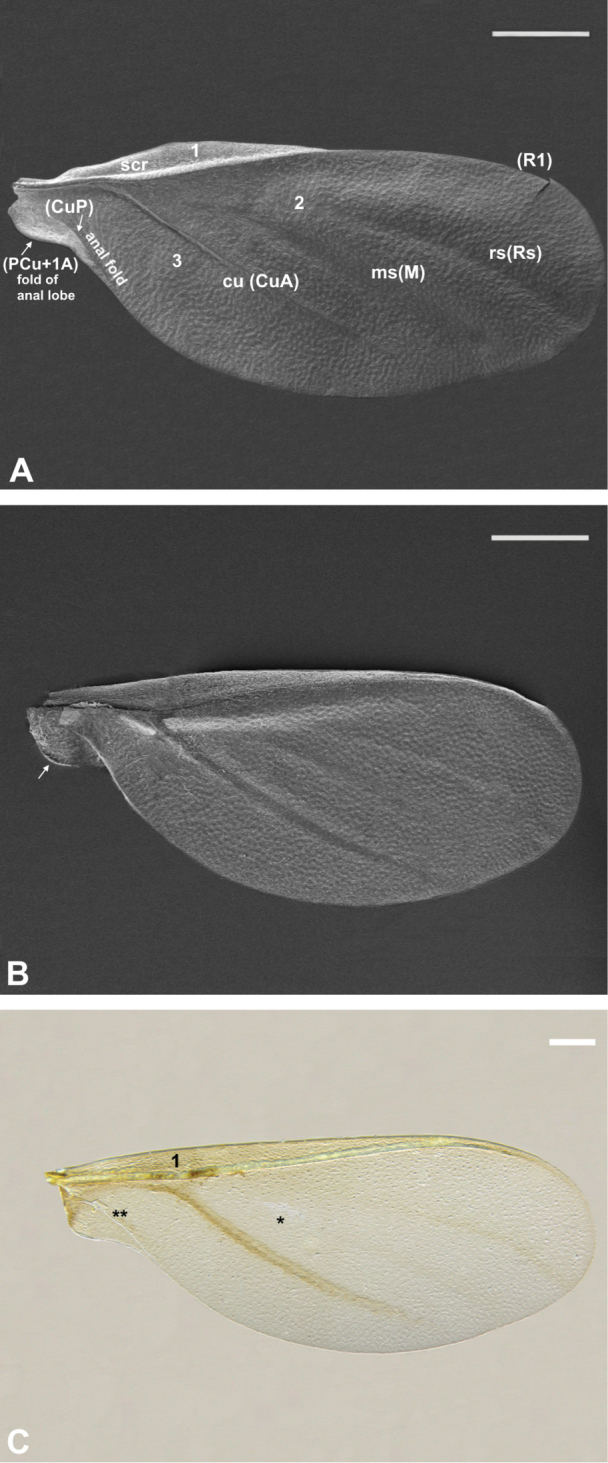
Forewing of *Orthezia urticae* (Linnaeus, 1758) **A** dorsal view under SEM **B** ventral view under SEM; Scale bar: 50 µm **C** dorsal view under LM; Scale bar: 250 µm; vein names without brackets after Koteja (1986), with brackets after Shcherbakov (2007); other symbols explained in the manuscript.

After Morrison (1925) and Schlee (1969), Koteja (1986) described wings in Ortheziidae as composed of five easily noticeable elements. These elements divide wing membrane into three fields (Koteja 1996) (1–3, Fig. 1A).

**Figure 2.**
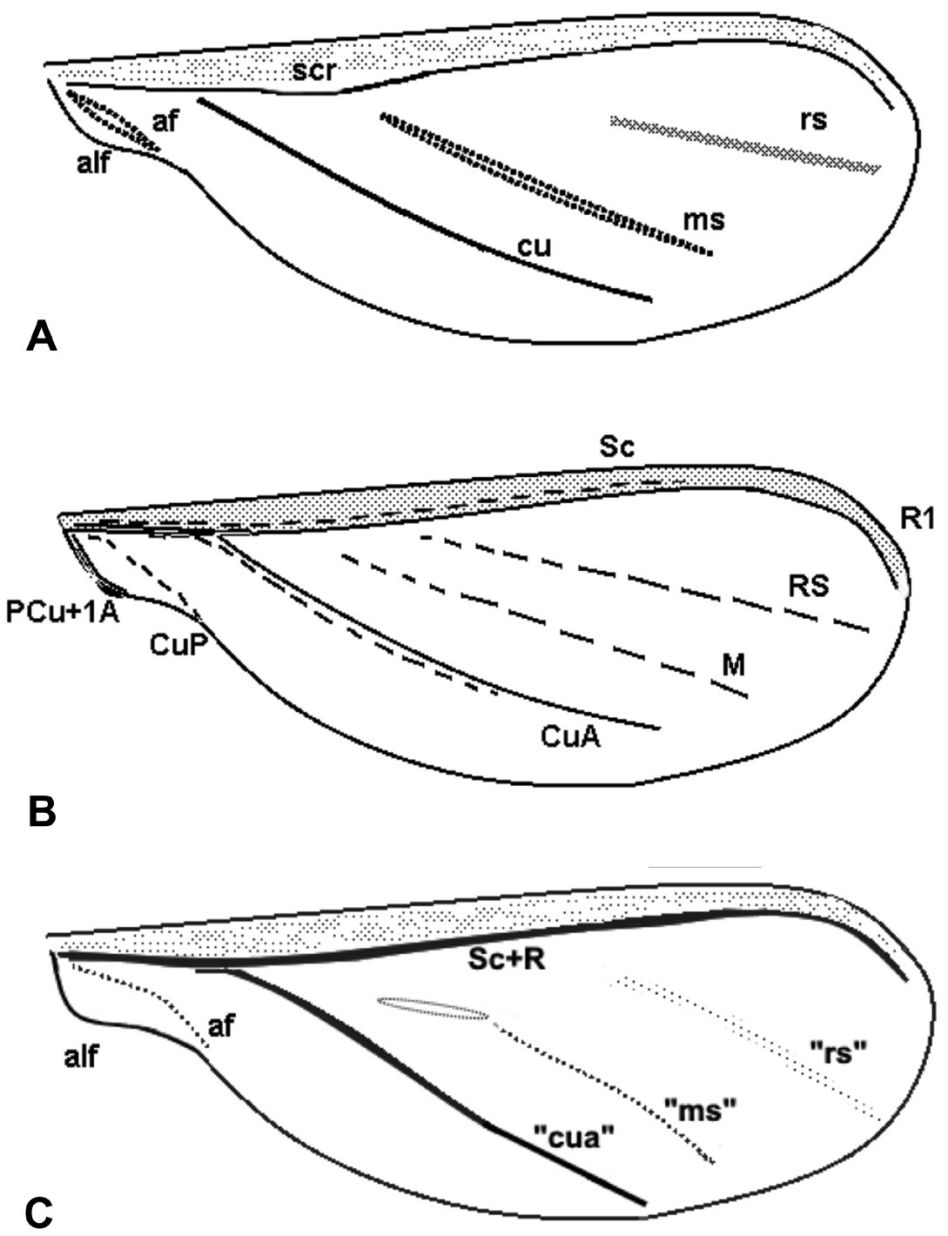
Schematic drawings of forewing of *Orthezia urticae* (Linnaeus, 1758) after **A** Koteja (1986) **B** Shcherbakov (2007) **C** present interpretation.

The first element, so-called Sc+R, is convex and composed of subcostal (Sc) and radial (R1) veins. Between the anterior wing edge and the subcostal ridge, there is a narrow field (1, Fig. 1A) called the costal field in most insects, and marginal thickening in coccoids. In the second field, the radial sector vein (Rs) (also known as the anterior diagonal vein) is visible as a long but weak patch. Below Rs, another vein is present – the medial sector vein (M) (= first diagonal clear line), which is also weak and long. These last two veins are placed in a triangular medial field (2, Fig. 1A). Another main vein on the wing membrane is a convex “basal diagonal vein” = cubital vein (Koteja 1986, Shcherbakov 2007 – Fig. 2A–B). Under this vein there is a transparent and concave part of the membrane (or shallow depression) called the anal fold contained within the third, posterior field of the wing (3, Fig. 1A).

To verify the above interpretations of the wing venation, we present the reconstruction of the course and the inner structure of wing veins in *Orthezia urticae* (Linnaeus, 1758). We also suggest changes in wing venation nomenclature (Fig. 2C; Table 1).

Recently, for the first time within Sternorrhyncha, cross-sections of wing were made for an aphid representative, *Aphis fabae* (Scopoli, 1763) (Franielczyk-Pietyra and Wegierek 2017). Thanks to that study it became possible to draw a broader comparison, which was an additional aim here.

**Table 1.** Wing vein nomenclature for *Orthezia urticae* (Linnaeus, 1758).

Author	Veins					
Koteja (1986)	scr	rs	ms	cu	–	–
Shcherbakov (2007)	Sc, R1	Rs	M	CuA	CuP	PCu+1A
Franielczyk-Pietyra et al. (2018)	Sc, R	“rs”	“m”	“cua”	–	–

## Materials and methods

### Scanning electron microscopy (SEM) and histology

**Figure 3.**
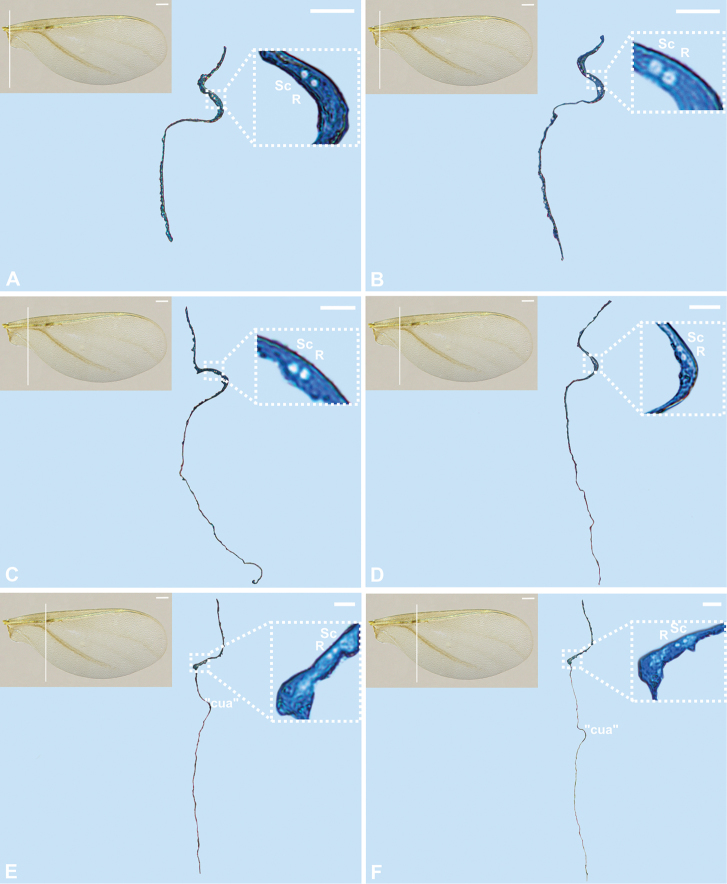
Cross-sections of the forewing of *Orthezia urticae* (Linnaeus, 1758); Scale bar: 50 µm. Scale bar of the forewing under light microscope 250 µm.

**Figure 4.**
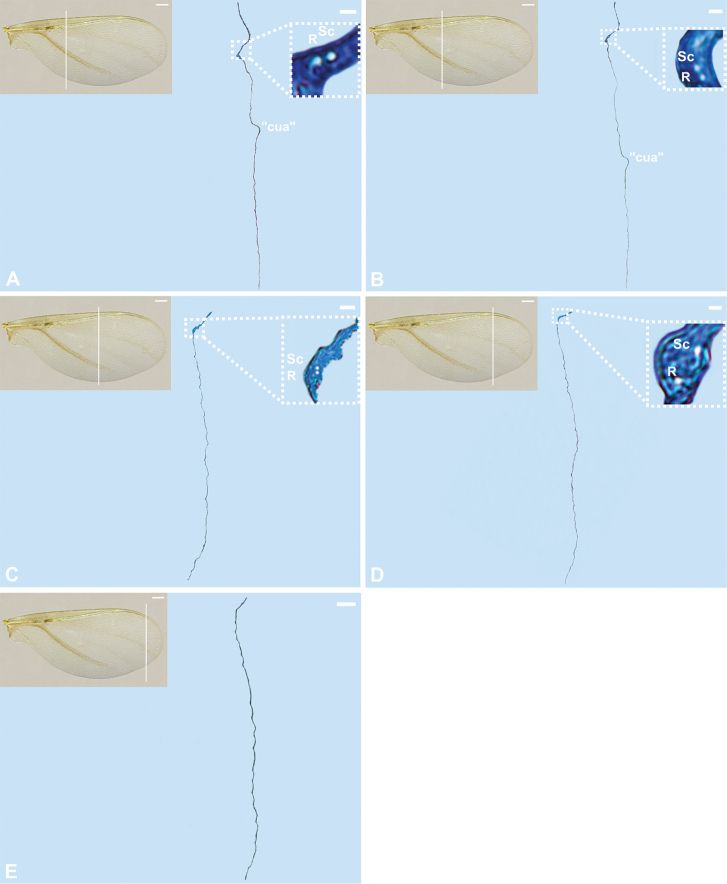
Cross-sections of the forewing of *Orthezia urticae* (Linnaeus, 1758); Scale bar: 50 µm. Scale bar of the forewing under light microscope 250 µm.

**Figure 5.**
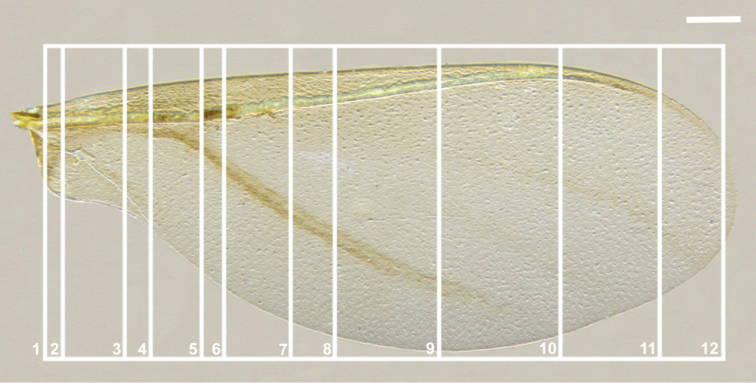
Light microscopy showing the forewing of *Orthezia urticae* (Linnaeus 1758), places of sectional cuts; Scale bar: 250 µm.

Methods were the same as in the previous article (Franielczyk-Pietyra and Wegierek 2017), both for SEM and histology. In the figures with cross-sections (Figs 3, 4) costal margin is at the top, anal margin at the bottom, upper surface to the right. Places of sectional cuts are presented in Fig. 5. Because of the delicacy of the wings, they were not cleaned and a large amount of wax is normal for this species.

Nomenclature of the veins is after Koteja (1986) and Shcherbakov (2007). The following abbreviations are used:

**af** anal fold;

**alf** fold of anal lobe;

**als** alar setae;

**cs** campaniform sensilla;

**R** radius;

**R1** first branch of radius (RA in the interpretation of Szwedo and Nel 2011);

“**rs” (= Rs)** radius sector (RP in the interpretation of Szwedo and Nel 2011);

“**ms” (= M)** media;

**mt** marginal thickening;

“**cua” (= CuA)** cubitus anterior;

**CuP** cubitus posterior;

**PCu+1A** fused postcubitus and first anal vein;

**Sc** subcostal;

**scr** subcostal ridge.

## Results

### Scanning electron microscopy

The dorsal surface shows a tuberculate sculpture formed by polygonal cells of cuticle. There are no microtrichia (Fig. 1A).

Subcostal ridge and cubital veins are convex, whereas medial and radial sectors look like small depressions. From the wing base to the place where CuA appears, the subcostal ridge is strongly convex and vertical, then the ridge turns closer to the cubital vein. It is still convex but not as much as at the beginning. Scr almost reaches the apex of the wing in that manner (Fig. 1A).

**Figure 6.**
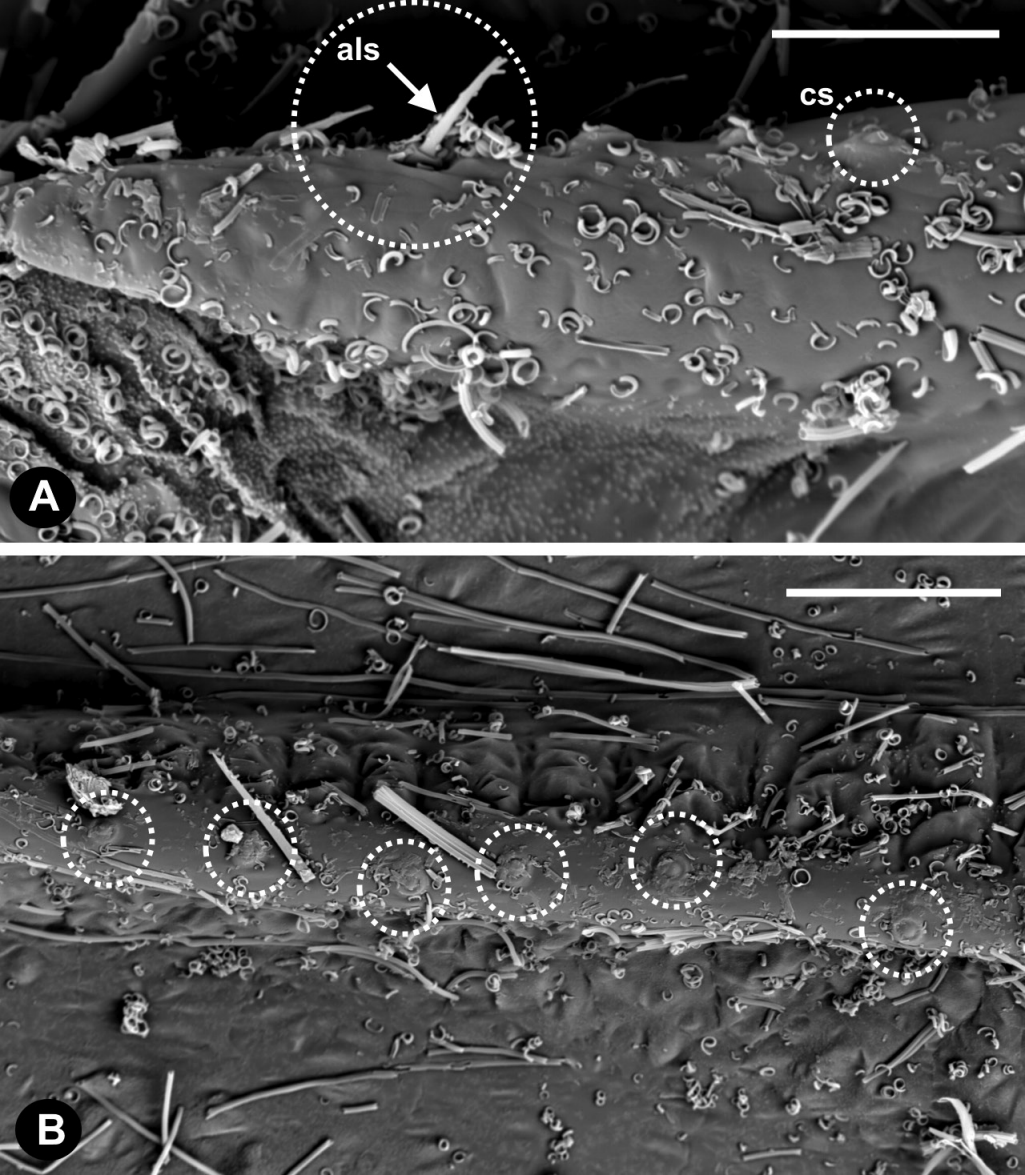
**A** Alar setae (als) and campaniform sensilla (cs) on the wing base of *Orthezia urticae* forewing; scale bar 20 µm **B** campaniform sensilla at the subcostal ridge. Scale bar: 25 µm.

There are a few small alar setae (als) at the beginning of subcostal ridge (Fig. 6A) and a row of about 30 campaniform sensilla (cs) (Fig. 6B).

Between the wing base and the concave anal fold there is a small anal lobe (Fig. 1A).

The ventral surface also shows a tuberculate sculpture (Fig. 1B). The whole course of scr and CuA veins is concave. Veins M and Rs look like a weak slightly convex patch. The proximal edge of anal lobe has a narrow fold to hold hamuli of the second pair of wings (white arrow, Fig. 1B).

### Light microscopy

Forewings are semitransparent and are elongate-oval in shape (Fig. 1C).

The subcostal ridge almost reaches the apex but turns towards the edge of the wing, so there is no pterostigma. No other vein reaches the wing apex. The costal field of the wing (1, Fig. 1C) is much more pigmented than other parts of the wing.

The cubital vein, which is strongly pigmented, does not seem to be directly connected with the subcostal ridge - it can be seen underneath as a separate element. The radial sector vein is less pigmented, and the medial vein is hardly visible. At the beginning of the latter, a small, narrow transparent patch is present (marked * in Fig. 1C).

The anal fold is a transparent element of the membrane (marked ** in Fig. 1C), visible as a thin line extending from the base of wing to the posterior margin of anal lobe.

The pterostigma is absent.

### Histology cross-sections

The wing membrane, at the beginning of sections, is thicker at the proximal part (Fig. 3A–E), then becomes thinner (Figs 3F, 4A–E). The subcostal ridge, inverted U-shaped on sections, comprises two veins inside (Sc and R). These are firstly placed at the base of scr (Fig. 3A–D). When the cubital vein appears, as mentioned earlier, scr turns closer to it. From this point the subcostal and radial veins run in scr closer to the triangular medial field (Fig. 3E). Both veins are present till the end of scr on cross-sections (Fig. 3A–4D). No nerves or tracheae are observed inside them. The cubital vein is present as a convex reinforcement of the wing membrane without any lumen inside (Figs 3E–F, 4A–B). The medial and radial sector veins are not visible on sections (Fig. 4C, D).

## Discussion

There is certainly no costal vein on cross-sections (which is equivalent to the lack of trachea). It is in contrast to aphid forewings, where a costal vein is present but there are no tracheae (Franielczyk-Pietyra and Wegierek 2017). This vein functions mainly as a reinforcement of the wing. In coccoids it was replaced by the subcostal ridge. The lack of costal trachea was regarded as a synapomorphy of Aphidomorpha and Coccomorpha by Shcherbakov (2007). According to the above results, this statement can be confirmed: neither costal vein nor costal trachea was observed.

The subcostal ridge, also known as the subcostal complex, comprises two veins – the subcostal and the radial ones, which run together from the wing base to the apex, where they turn towards the wing margin and disappear. Cross-sections confirm the theory of Koteja (1986) and confirm that these veins can be considered to have lumens which create tunnels for haemolymph. The subcostal complex can be regarded as a common stem for these two veins, which is similar to the situation in *Aphis fabae* (Franielczyk-Pietyra and Wegierek 2017). However, in aphids the common stem is composed of radius, media and cubital anterior veins as well as their tracheae. Besides the content of the common part, the difference is reflected in the fact that in coccoids both veins run together to the wing apex, while in aphids R, M and Cu veins spread apart.

The presence of the subcostal vein is note-worthy because it is reported for the first time in a representative of the Sternorrhyncha. Neither aphids (Franielczyk-Pietyra and Wegierek 2017) nor psyllids (Franielczyk-Pietyra and Wegierek unpubl. data) have this vein. This brings coccoids closer to the Naibiidae family, which is considered as ancestral to true scale insects (Shcherbakov 2007).

**Figure 7.**
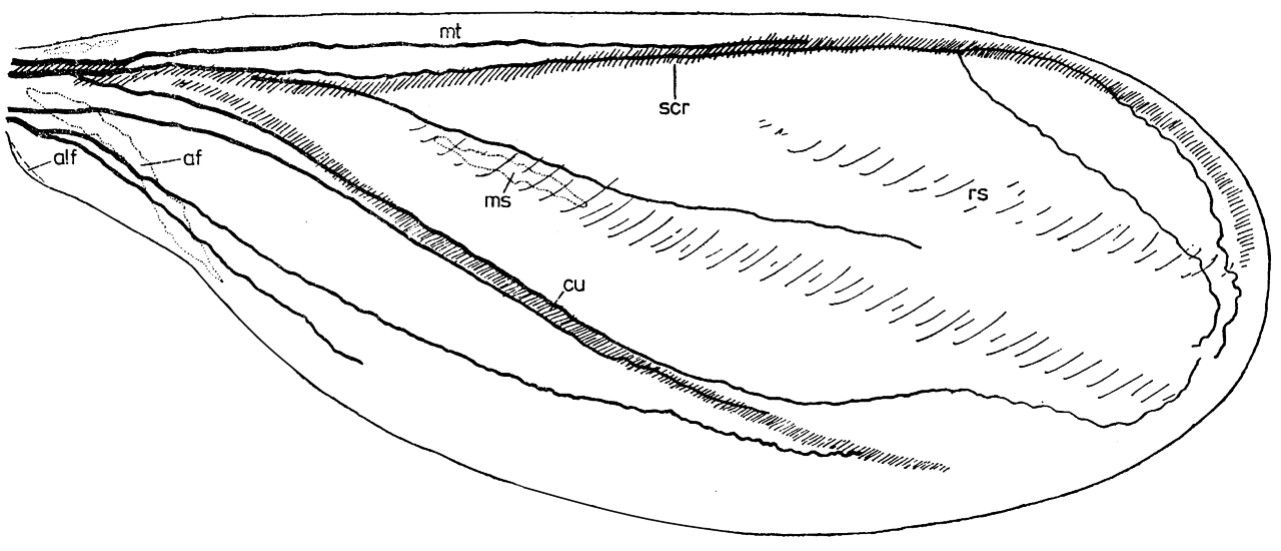
Tracheal system of forewing of *Orthezia urticae* (Linnaeus 1758) after Koteja (1986).

**Figure 8.**
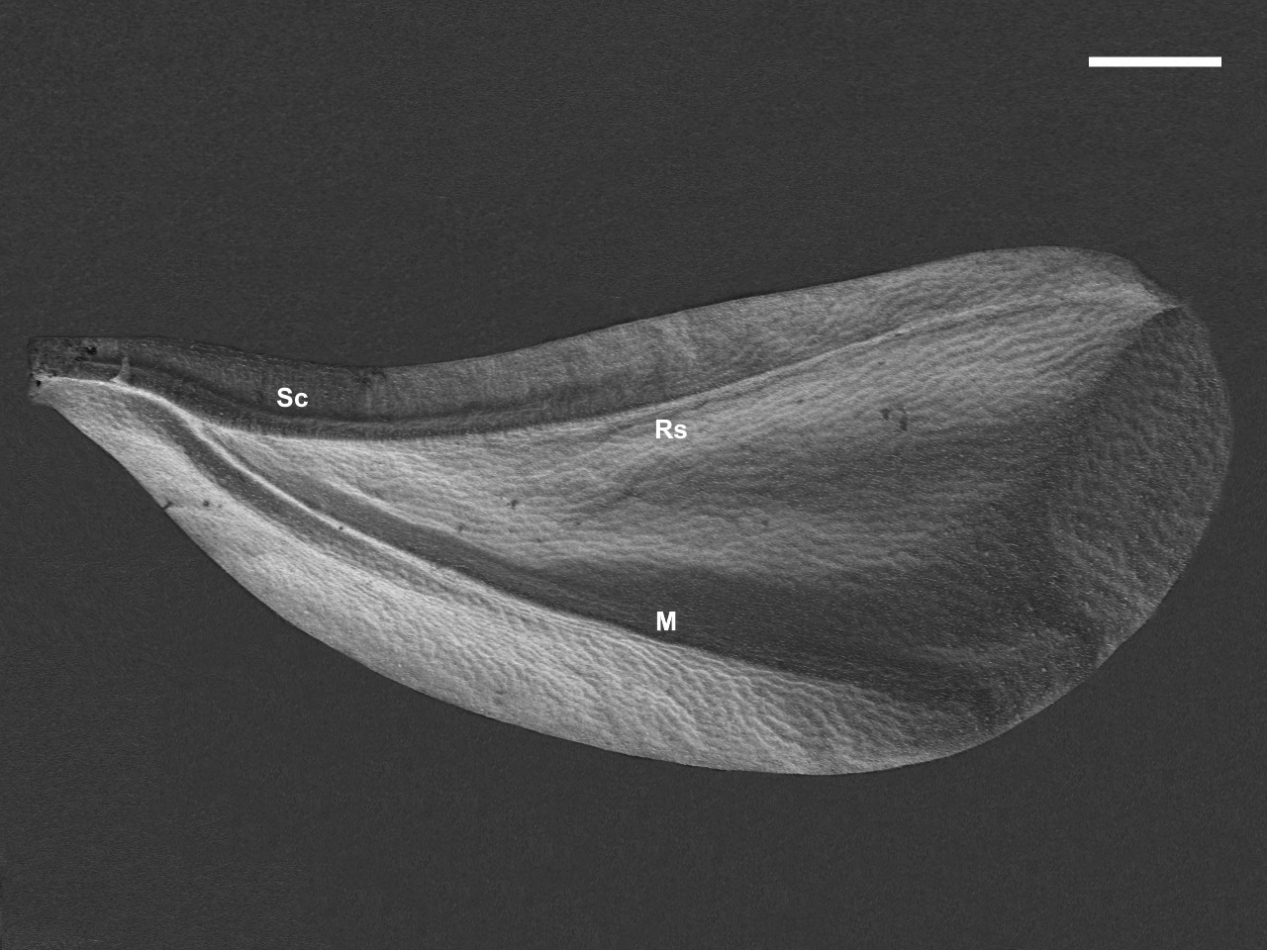
Forewing of *Planococcus citri* (Risso 1813) under SEM. Scale bar: 100 µm.

Darker parts of the membrane, regarded as medial and radial sector veins, are only more pigmented (sclerotized) parts of the wing. Probably they are remnants of veins, as evidenced by Koteja (1986). The tracheal system which he drew is more complex than the system of veins (Fig. 7). So far it has been believed, that Patch (1909) in her work showed tracheal and vein systems for all coccoids. However, two coccoid species studied by her, *Planococcus citri* (Risso 1813) (= *Pseudococcus citri*) (Pseudococcidae) (Fig. 8) and an unidentified species of *Dactylopius* Costa, 1829 (Dactylopiidae), belong to the group of neococcoids. Also, the only visible veins on those wings are called Sc, Rs and M by Patch. Judging by the course of the tracheae in the presented neococcoid wing, the medial vein should probably be considered as the cubital anterior, and the radial sector as the radial vein (because of no further branching). As there is no trace of other veins, the reduction of venation proves to have taken place in more advanced species. It is the reason why Rs and M should not be called veins but only slight depressions or transparent patches and marked as “rs” and “m”.

In aphids, radial sector and medial veins are very clearly outlined and in *A. fabae* the latter is divided into three branches: M_1_, M_2_ and M_3+4_ (Franielczyk-Pietyra and Wegierek 2017); it additionally stiffens the wing, but these veins are not equally well developed in all aphids. Some have media with two branches or unbranched.

Additionally, Shcherbakov (2007) described Rs and M as concave veins in coccoids, which cannot be confirmed, as it is only pigmentation. Also, he claimed that veins Sc+R and CuA are twofold; both contain convex and concave parts. This idea may have been drawn from the shadow formed in SEM under a strongly convex subcostal ridge and the element called the cubital vein. Histological methods did not confirm this. Moreover, two cubitus anterior veins (CuA_1_, CuA_2_) are distinctive of aphids. Atrophy of this veins in the coccoids forewing is probably associated with the existence of the anal lobe and with hindwing diminution (Shcherbakov 2007).

The same author indicated the presence of PCu+1A vein on the edge of the anal lobe, as a synapomorphy for both aphids and coccoids. Likewise, Koteja (1986) described the presence of the anal vein as a common feature for the Ortheziidae and most archaeococcoids (absent in neococcoids). Both authors were probably referring to the same vein, PCu+1A. However, its presence cannot be confirmed, due to the fact that neither aphids (Franielczyk-Pietyra and Wegierek 2017), nor scale insects (coccoids) show such an element in cross-sections. Probably this vein is moved to the anal wing margin. In coccoids the wing anal lobe is always present in species with halteres (Giliomee 1967). In such species the edge of the anal lobe has a narrow fold on the ventral surface, the place where hamuli are attached (two hooked setae from halteres) (Koteja 1986). The wing anal lobe has not been described in aphids; they have two similar pairs of wings linked together by a wing-coupling apparatus (Franielczyk-Pietyra and Wegierek 2017). As suggested by Koteja (1996), the occurrence of a connection between both wings could be regarded as synapomorphy of aphids and coccoids.

No archaeococcoids have developed microtrichia, which are characteristic of the wing surface of neococcoids (Koteja 1996). It explains the absence of these structures on the wings examined.

Alar setae and campaniform sensilla observed at the subcostal ridge (Fig. 6) confirm data presented by Koteja (1986), and the number is congruent with his opinion. According to this author, 3–8 alar setae are located on the subcostal ridge near the wing base, whereas campaniform sensilla create a row of 25–43 setae along the subcostal ridge. Our observations make it possible to state that alar setae do occur, but their number is hard to estimate because of their fragility. Due to the fact that there is a very dense wax covering, some sensilla may not be clearly visible, so it is not easy to count them correctly. We counted about 30 of them. As campaniform sensilla are the only example of mechanoreceptors known to be involved in flight stabilization (Chapman 2013), their number and placement is understandable. These sensilla are distributed along the subcostal ridge to take on the role of non-existing veins. A recent study of campaniform sensilla in the aphid genus *Mindarus* (Montagano & Favret, 2016) revealed fewer elements on the forewing. It is probably associated with frequent wing flexing and different distribution of sensilla on aphid forewings in comparison with scale insects.

The hypodermal club-shaped thickening (structure similar to pterostigma in other insects) without any veins around (Koteja 1996; Koteja and Azar 2008), is known only in some coccoids within the Margarodidae family *sensu lato.* Therefore, the lack of this structure in our results is not surprising.

Comparing forewings in aphids and coccoids, we can conclude that the elements distinctive of coccoids include only two veins, subcostal and radial ones in the common stem and the presence of the wing anal lobe (Table 2). It seems that this very delicate structure in coccoid representatives is related to the short life span of scale insect males (Koteja 1985).

Based on morphological (Hennig 1981; Carver, Gross, Woodward 1991) and molecular studies (von Dohlen and Moran 1995; Xie et al. 2008), aphids and coccoids are considered as sister groups. The results summarized in Table 2 do not show many affinities between the forewings in these groups. This only indicates that wing venation may not be a good character for the analysis of similarities or phylogeny.

Due to the fact that histological methods revealed only two veins, the subcostal and the radial ones in the subcostal ridge, it seems necessary to change the nomenclature of wing veins for *Orthezia urticae* Linnaeus 1758. The element known as cubitus anterior vein should be regarded as a convex sclerotized part used only to strengthen the wing and should be marked as “cua”. Two other elements, known as radial sector and medial sector veins, should be marked as “rs” and “m”, but not considered any longer as veins.

**Table 2.** Comparison of the forewing structures between a representative of aphids and of coccoids.

Structure	*Aphis fabae*	*Orthezia urticae*
C	+	–
Sc	–	+
R	+	+
R_1_	+	–
Rs	+	„rs”
M	+	„m”
M_1_	+	–
M_2_	+	–
M_3+4_	+	–
CuA	+	„cua”
CuA_1_	+	–
CuA_2_	+	–
pterostigma	+	–
common stem	R+M+CuA	Sc+R
anal lobe	–	+
